# Supplementary material for: DAFuzz: data-aware fuzzing of in-memory data stores
Source: PeerJ Comput Sci. 2023 Sep 19;9:e1592. doi: 10.7717/peerj-cs.1592 (PMC10557509; doi:10.7717/peerj-cs.1592)
Supplement: Supplemental Information 2 [file peerj-cs-09-1592-s002.zip › DAFuzz/experimental/canvas_harness/canvas_harness.html]

## Results
