# Supplementary figures and images for: DAFuzz: data-aware fuzzing of in-memory data stores
Source: PeerJ Comput Sci. 2023 Sep 19;9:e1592. doi: 10.7717/peerj-cs.1592 (PMC10557509; doi:10.7717/peerj-cs.1592)

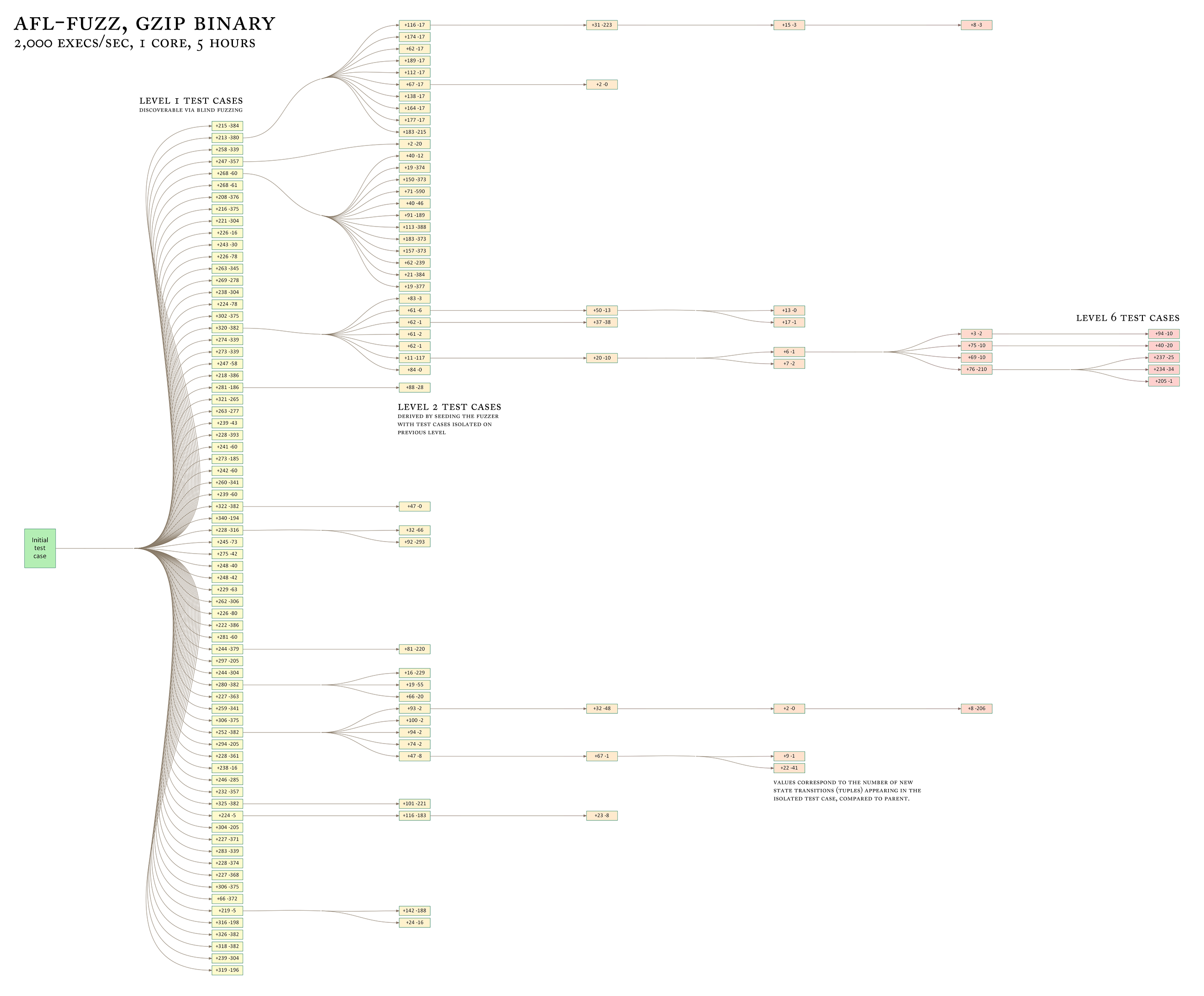

Supplement: Supplemental Information 2 [file peerj-cs-09-1592-s002.zip › DAFuzz/docs/visualization/afl_gzip.png]

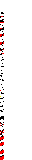

Supplement: Supplemental Information 2 [file peerj-cs-09-1592-s002.zip › DAFuzz/docs/vuln_samples/firefox-gif-leak2.gif]

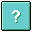

Supplement: Supplemental Information 2 [file peerj-cs-09-1592-s002.zip › DAFuzz/testcases/images/bmp/not_kitty.bmp]

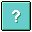

Supplement: Supplemental Information 2 [file peerj-cs-09-1592-s002.zip › DAFuzz/testcases/images/gif/not_kitty.gif]

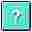

Supplement: Supplemental Information 2 [file peerj-cs-09-1592-s002.zip › DAFuzz/testcases/images/jpeg/not_kitty.jpg]

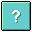

Supplement: Supplemental Information 2 [file peerj-cs-09-1592-s002.zip › DAFuzz/testcases/images/png/not_kitty.png]

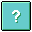

Supplement: Supplemental Information 2 [file peerj-cs-09-1592-s002.zip › DAFuzz/testcases/images/png/not_kitty_alpha.png]

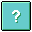

Supplement: Supplemental Information 2 [file peerj-cs-09-1592-s002.zip › DAFuzz/testcases/images/png/not_kitty_gamma.png]

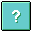

Supplement: Supplemental Information 2 [file peerj-cs-09-1592-s002.zip › DAFuzz/testcases/images/png/not_kitty_icc.png]

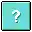

Supplement: Supplemental Information 2 [file peerj-cs-09-1592-s002.zip › DAFuzz/testcases/images/webp/not_kitty.webp]
